# Estimated glomerular filtration rate in Korean patients exposed to long-term lithium maintenance therapy

**DOI:** 10.1186/s40345-022-00249-5

**Published:** 2022-02-07

**Authors:** Yunji Cho, Dongbin Lee, Ji Hyun Baek, Kyung Sue Hong

**Affiliations:** 1 Department of Psychiatry, Samsung Medical Center, Sungkyunkwan University School of Medicine, 81, Irwon-ro, Gangnam-gu, Seoul, 06351 Korea; 2 Department of Digital Health, Samsung Medical Center, Samsung Advanced Institute for Health Sciences and Technology (SAIHST), Sungkyunkwan University, Seoul, Korea; 3 Samsung Biomedical Research Institute, Seoul, Korea

**Keywords:** Bipolar disorder, Lithium, Valproate, Estimated glomerular filtration rate, Renal function, Lithium-induced nephrotoxicity, Chronic kidney disease

## Abstract

**Background:**

Lithium-induced nephrotoxicity has long been debated. However, it has been rarely explored in Asian populations. The aim of the present study was to assess the effect of lithium maintenance therapy on estimated glomerular filtration rate (eGFR) in Korean patients diagnosed with a psychiatric illness.

**Methods:**

This was a single-centered, retrospective study that included patients treated with lithium or comparator drug (valproate) in Samsung Seoul Medical Center between November 1994 and July 2020. Patients diagnosed with ICD codes F20-33 who had  ≥ 6 months of exposure to lithium or valproate were included. Patients had to have  ≥ 1 baseline and  ≥ 2 post-baseline eGFR data with post-baseline data having an interval of at least 30 days. Chronic kidney disease (CKD) was defined as CKD stage 3 (eGFR  < 60 mL/min/1.73^2^). To be considered as CKD, the threshold had to be met at two consecutive post-baseline measurements. Those treated with both lithium and valproate, diagnosed with CKD stages 3–5, diagnosed with a renal disease, or received kidney transplantation were excluded.

**Results:**

A total of 766 patients were included (242 treated with lithium and 524 with valproate). Two (0.8%) in the lithium group and 8 (1.5%) in the valproate group developed CKD stage 3. None developed CKD stages 4–5. Median yearly eGFR change was − 1.3 mL/min/1.73^2^ (IQR: − 6.8, 1.7) for the lithium group and − 1.1 mL/min/1.73^2^ (IQR: − 4.5, 1.5) for the valproate group, showing no significant difference between the two groups (*p * = 0.389). The rate of decline was more rapid for those with CKD in both groups. eGFR values of lithium and valproate groups did not show significant differences during a follow-up duration of 15 years or more. A significant negative correlation between baseline eGFR and yearly eGFR change was identified in a linear regression analysis.

**Conclusions:**

In Korean patients, treatment with lithium did not increase the risk of developing CKD compared to treatment with valproate. Prevalence of CKD was lower than those previously reported in western populations. Low baseline eGFR showed significant correlation with changes in renal function.

**Supplementary Information:**

The online version contains supplementary material available at 10.1186/s40345-022-00249-5.

## Background

Lithium is one of the most established drugs in long-term treatment of bipolar disorder. It is effective in preventing relapses of mood episodes and in reducing risk of suicide (Geddes and Miklowitz 2013). Because of its narrow therapeutic index, clinicians are required to regularly monitor serum lithium levels and to titrate the dosage accordingly. Concerns have been raised regarding nephrotoxic effects of long-term lithium maintenance, with prior studies in the 1970’s reporting structural tubular damage and interstitial fibrosis in biopsy findings of lithium users (Hestbech et al. 1977). Following studies have confirmed such morphologic findings after examining tubulointerstitial nephropathy and relative preservation of the glomeruli (Hansen et al. 1979; Aurell et al. 1981).

Since then, the effect of long-term lithium treatment on renal function has been the topic of debate. Several studies have reported decline of renal function after lithium use, while others have reported negative associations between the two (McKnight et al. 2012; Bocchetta et al. 2015; Clos et al. 2015). Lithium-induced nephropathy has been recognized to be slow in progression (“creeping creatinine”). Its rate of progression is associated with age, treatment duration, cumulative dose, and episodes of toxicity (Presne et al. 2003; Aiff et al. 2015; Bocchetta et al. 2015; Clos et al. 2015). Compared to other mood stabilizers and atypical antipsychotics, lithium is associated with decline in renal function (eGFR  < 60 mL/min/1.73^2^), but to a lesser degree with greater eGFR declines (eGFR  < 30 mL/min/1.73^2^) (Hayes et al. 2016). Incidence of CKD has been reported to be 21–55% in long-term lithium users (Tredget et al. 2010). Progression to end-stage renal disease (ESRD) has been found to be uncommon (0.5–1%) and to require longer time to develop (Lepkifker et al. 2004; Bendz et al. 2010; Tredget et al. 2010; Aiff et al. 2014). Other reports have stated that the rate of eGFR decline does not significantly differ between long-term lithium users and patients treated with other psychotropic agents, advocating the safety of lithium maintenance therapy (Clos et al. 2015). These discrepancies might have derived from differences in study design and comparator groups. As patients who are exposed to lithium have a high probability of co-prescription of other psychotropic medication (Baek et al. 2014; Fung et al. 2019), additional effects of polypharmacy on renal function should be minimized by selecting comparison groups who are likely to be on similar sets of medications. Previous findings based on long-term observational data should also be reviewed with consideration of several biases, including ascertainment bias, channeling bias and survival bias. Increased risk of renal impairment associated with lithium treatment reported in numerous studies may be a result of overestimation due to surveillance bias (Nielsen et al. 2018).

Lithium-induced nephrotoxicity is still a topic of controversy even after a long history of debate. Very limited research exists regarding lithium’s effect on renal function in an Asian population. Thus, the aim of the present study was to assess the effect of lithium maintenance therapy on renal function represented by eGFR in a sample of Korean patients diagnosed with a psychiatric illness. We included all patients who were chronically exposed to lithium in a tertiary care setting hospital in Korea and set the comparison group as those who were exposed to valproate.

## Methods

### Study participants

This study was designed as a single-centered, retrospective study that included patients who had records of being treated with lithium in the Department of Psychiatry, Samsung Seoul Medical Center, Korea between November 9, 1994 and July 20, 2020. Patients who had been treated with valproate, one of first-line drugs in the treatment of bipolar disorder, were assigned to the comparator group. Patients eligible for inclusion were those aged 18 years or older at baseline (the first day of lithium or valproate prescription), those who had at least 6 months of exposure to either lithium or valproate, and those who had  ≥ 1 baseline and  ≥ 2 post-baseline eGFR data (two consecutive post-baseline data requiring at least 30 days of interval). This study included patients who had ICD codes of F20-29 (schizophrenia or psychotic disorders), F30-31 (bipolar disorders), and F32-33 (depressive disorders) as main diagnosis. Those with other psychiatric and medical conditions as main diagnosis, including brain tumor related conditions (ICD codes C70-71) and epilepsy (ICD codes G40-41), were not included. It is to be noted that the Korean health insurance system is based on a claims data generated by healthcare providers for reimbursement purposes (Kim et al. 2017). As such, certain ICD codes are necessary for coverage of specific drug prescriptions or eligibility for healthcare provision. Therefore, discrepancies can occur between diagnoses entered in the data system and diseases that a patient actually has.

Those who had been treated with both lithium and valproate, either at different time or at the same time, were excluded from the analysis. They were identified through prescription records and serum therapeutic drug monitoring (TDM). Those with baseline eGFR  < 60 mL/min/1.73^2^ (CKD stages 3–5), those with a baseline kidney disease (ICD codes N00-08 and N10-19), and those who had received kidney transplantation (ICD codes T86.1 and Z94.0) were also excluded.

This study was approved by the Institutional Review Board of Samsung Seoul Medical Center (IRB no. 2020-12-029-001). The requirement for written informed consent was exempt by the IRB because this study was based on an anonymized dataset of electronic health records and lab results.

### Definitions

CKD was defined as eGFR  < 60 mL/min/1.73^2^ (CKD stage 3). The threshold had to be met at the two most recent post-baseline eGFR data measured at least 30 days apart. eGFR was calculated with the Chronic Kidney Disease Epidemiology Collaboration (CKD-EPI) equation, which took age, sex, and race into account (Levey et al. 2009). Baseline eGFR was defined as the eGFR value measured before initiation of lithium or valproate use. Baseline diagnosis of hypertension was confirmed using ICD-9 codes 401–405 and ICD-10 codes I10–16 before prescription of lithium or valproate. Baseline diabetes mellitus was defined as ICD-9 codes 250.0–250.9 and ICD-10 codes E08–13 recorded before the use of lithium or valproate.

We used three different cutoff points in defining episodes of lithium toxicity: lithium TDM  > 0.8 mmol/L, TDM  > 1.0 mmol/L, and TDM  > 1.2 mmol/L. Lithium has a narrow therapeutic index of 0.5–0.8 mmol/L in maintenance phase and 0.8–1.2 mmol/L in acute manic phase. It has been reported that even one serum level of  > 1.0 mmol/L can cause significant effect on eGFR (Raja 2011; Kirkham et al. 2014). Thus, cutoff points were set at short intervals to closely monitor effects of toxic lithium levels.

### Statistical analyses

Clinical characteristics were compared between those treated with lithium or valproate presented as mean  ±  standard deviation (SD) or as number (%). Median years to reach eGFR  < 60 mL/min/1.73^2^ and yearly eGFR change were calculated for each group (lithium group and valproate group).

We prepared a scatter plot of eGFR over the follow-up period and compared the overall trend in eGFR change between the lithium group and the valproate group. Linear regression was carried out with yearly eGFR change as dependent variable to explore associations between demographic factors, baseline comorbidities, and clinical variables such as medication of use (lithium vs. valproate), primary diagnosis, baseline eGFR, treatment duration, average daily dose of medication and lithium toxicity. Kaplan–Meier method was used to predict the number of years required to reach eGFR  < 60 mL/min/1.73^2^. All statistical analyses were performed using R 4.0.3. Statistical significance was considered when *p* value was less than 0.05.

## Results

**Fig. 1:**
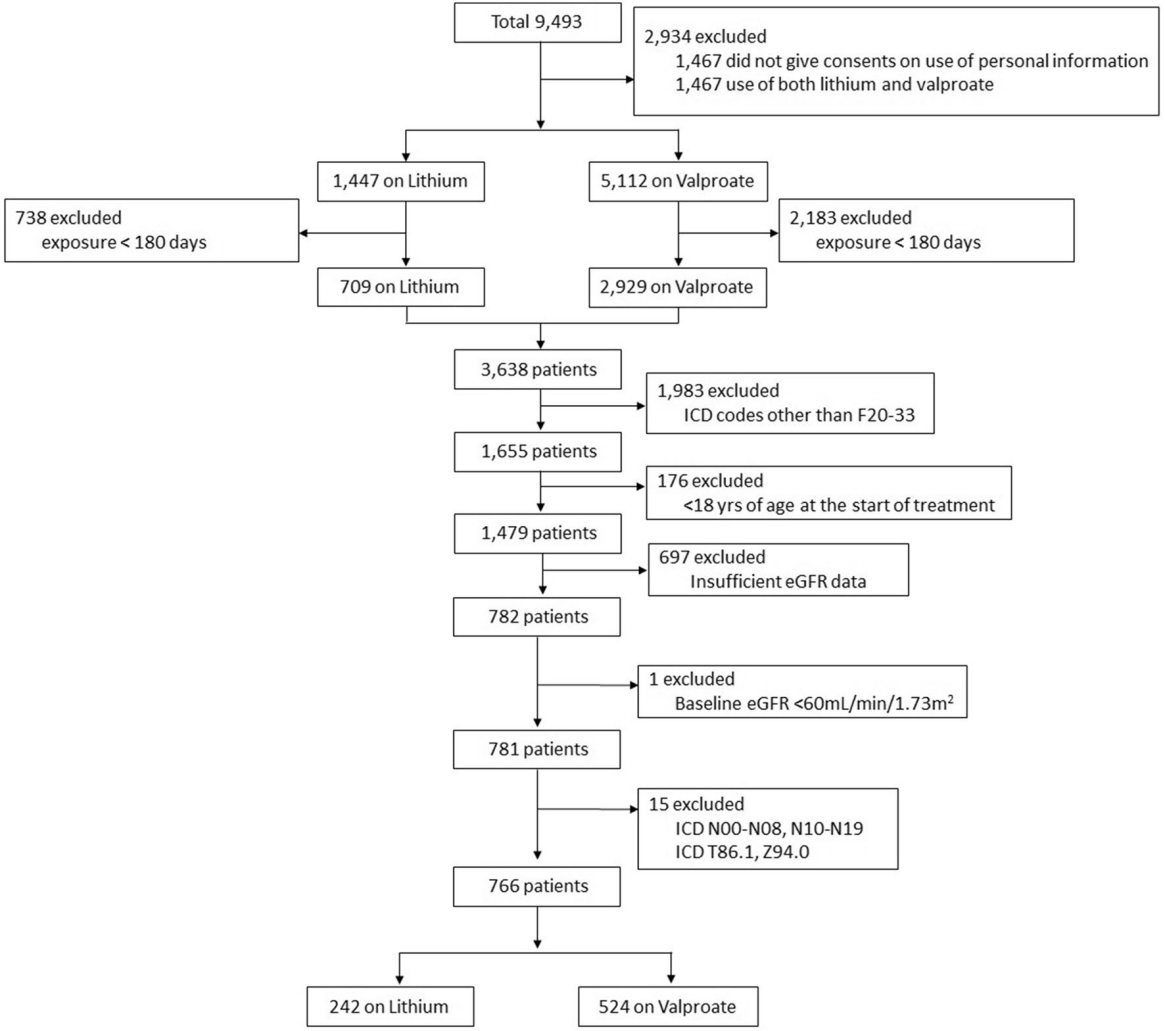
Inclusion of the study group

Among 9493 patients with at least one prescription record of lithium or valproate during the observation period, 766 patients (242 on lithium and 524 on valproate) met the inclusion and exclusion criteria (Fig. 1). Mean age of the study population was 39.3 years (SD: 16.0 years; median: 35.0 years; range: 18.0–86.0 years). A total of 410 (53.5%) women and 356 (46.5%) men were included. Of them, 425 (55.5%) patients had primary ICD-10 diagnosis of bipolar disorder (codes F30–31).

Table 1 shows baseline characteristics of the study population. Patients treated with lithium tended to be younger than those treated with valproate (mean age: 37.0 years vs. 40.4 years, *p * = 0.005). No significant differences existed between the two groups regarding sex and baseline comorbidities of hypertension and diabetes mellitus. As for primary psychiatric diagnoses, the lithium group included a larger proportion of those diagnosed with bipolar disorder (ICD-10 codes F30–31) than the valproate group (71.5 vs. 48.1%).

**Table 1:** Baseline characteristics of subjects

	Lithium (n = 242)	Valproate (n = 524)	*p*
Age	37.0 ± 14.6	40.4 ± 16.5	0.005
Sex (%)			
Women	134 (55.4)	276 (52.7)	0.536
Men	108 (44.6)	248 (47.3)	
Baseline hypertension (%)	23 (9.5)	42 (8.1)	0.605
Baseline diabetes (%)	10 (4.1)	17 (3.2)	0.683
Primary psychiatric diagnoses, ICD-10 (%)			
F20–29 (schizophrenia or psychotic disorders)	40 (16.5)	115 (21.9)	< 0.001
F30–F31 (bipolar disorders)	173 (71.5)	252 (48.1)	
F32–F33 (depressive disorders)	29 (12.0)	157 (30.0)	
eGFR < 60 mL/min/1.73 m^2^ (%)	2 (0.8)	8 (1.5)	0.733
Follow-up duration (year)	4.2 ± 4.2	5.3 ± 5.2	0.002
Cumulative years on lithium treatment	3.5 ± 3.5	–	–
Cumulative years on valproate treatment	–	4.3 ± 4.2	–
Average TDM			
Lithium TDM	0.6 ± 0.2	–	
Valproate TDM	–	56.7 ± 20.5	
Episodes of lithium toxicity (≥ 1 episode)			
Lithium TDM > 0.8 mmol/L (%)	112 (46.3)	–	–
Lithium TDM > 1.0 mmol/L (%)	54 (22.3)	–	–
Lithium TDM > 1.2 mmol/L (%)	17 (7.0)	–	–
Daily dosing schedule^a^			
Lithium once-daily dosing	0.9 ± 0.2	–	–
Valproate once-daily dosing	–	0.8 ± 0.4	–
Yearly median eGFR change (mL/min/1.73 m^2^)	− 1.3 (IQR − 6.8, 1.7)	1.1 (IQR − 4.5, 1.5)	0.389
Baseline eGFR (mL/min/1.73 m^2^)	103.9 ± 18.2	103.1 ± 18.4	0.533
Last eGFR (mL/min/1.73 m^2^)	100.9 ± 18.4	100.6 ± 19.0	0.851
Second to last eGFR (mL/min/1.73 m^2^)	101.9 ± 18.5	101.3 ± 18.2	0.629
Average eGFR values by year (mL/min/1.73 m^2^)			
Year 1 eGFR	104.6 ± 16.9	103.8 ± 18.1	0.549
Year 3 eGFR	104.8 ± 15.6	101.4 ± 17.6	0.208
Year 5 eGFR	99.5 ± 17.2	102.1 ± 17.7	0.507
Year 7 eGFR	98.0 ± 16.1	98.0 ± 16.2	0.995
Year 10 eGFR	96.4 ± 15.7	96.9 ± 18.1	0.945
Year 12 eGFR	95.2 ± 11.5	96.5 ± 15.9	0.842
Year 15 eGFR	73.7 ± 21.1	99.6 ± 15.6	0.085
Year 20 eGFR	–	81.5 ± 21.9	–

^a^Average proportion of once-daily dosing per patient

The valproate group had a higher percentage of patients whose eGFR fell below 60 mL/min/1.73 m^2^ (CKD stage 3) during the follow-up period, although the difference between the two was insignificant [2 (0.8%) in the lithium group vs. 8 (1.5%) in the valproate group, *p * = 0.733]. No patient in either group reached CKD stages 4–5.

Patients treated with valproate had a longer follow-up duration [4.2 years for the lithium group (SD: 4.2 years) vs. 5.3 years (SD: 5.2 years) for the valproate group, *p * = 0.002]. Mean cumulative duration of lithium exposure was 3.5 years (SD: 3.2 years; median: 2.4 years; range, 0.5–21.0 years), while mean cumulative duration of valproate treatment was 4.3 years (SD: 4.2 years; median: 3.1 years; range: 0.5–24.6 years). The average serum lithium level was 0.6 mmol/L (SD: 0.2) and the average serum valproate level was 56.7 μg/mL (SD: 20.5).

The lithium group had a median yearly eGFR change of − 1.3 mL/min/1.73 m^2^ (IQR: − 6.8, 1.7) and the valproate group had a median yearly eGFR change of − 1.1 mL/min/1.73 m^2^ (IQR: − 4.5, 1.5). There was no significant difference in yearly eGFR change between lithium and valproate groups (*p * = 0.389). Baseline and yearly eGFR values showed no significant differences between the two groups up to year 15, at which the mean eGFR was 73.7 ± 21.1 mL/min/1.73 m^2^ for the lithium group and 99.6 ± 15.6 mL/min/1.73 m^2^ for the valproate group, showing no significant (*p*  = 0.085) difference between the two.

Among patients who reached CKD stage 3, median years to reach eGFR  < 60 mL/min/1.73 m^2^ were 8.7 years (range, 4.9–21.4 years) for the overall study population, 12.8 years (range, 9.8–15.9 years) for the lithium group, and 7.4 years (range, 4.9–21.4 years) for the valproate group.

Two lithium users who developed CKD stage 3 both had baseline diagnosis of hypertension with a history of lithium toxicity reaching serum level  > 1.0 mmol/L during follow-up. Each of them required 15.88 years and 9.82 years since the start of lithium until development of CKD (Additional file 1: Table S1).

Linear regression revealed significant negative correlation between baseline eGFR and yearly eGFR change (Table 2, adjusted coefficient: − 2.2; *p*  = 0.030). The choice of drug (lithium vs. valproate) did not display significant correlation with eGFR change in linear regression analyses. Subgroup analyses by diagnoses did not show significant correlations with annual eGFR decline, with the exception of age in patients diagnosed with F32–33 (Additional file 1: Tables S2-1, S2-2, S2-3).

**Table 2:** Linear regression predicting yearly eGFR change

	Crude coefficient (95% CI)	Crude *p*	Adjusted coefficient (95% CI)	Adjusted *p*
Age	− 0.26 (− 1.94, 1.42)	0.764	− 2.39 (− 4.84, 0.05)	0.056
Sex (men vs. women)	− 13.37 (− 66.81, 40.07)	0.624	− 20.04 (− 75.92, 35.84)	0.482
Baseline hypertension	− 11.69 (− 107.43, 84.05)	0.811	− 7.87 (− 112.71, 96.96)	0.883
Baseline diabetes	− 0.47 (− 145.63, 144.69)	0.995	25.83 (− 128.75, 180.41)	0.743
Primary psychiatric diagnoses, ICD-10 (ref. = F20–29)				
F30–31	− 16.45 (− 85.42, 52.53)	0.640	− 14.45 (− 86.41, 57.52)	0.694
F32–33	53.15 (− 27.04, 133.35)	0.194	62.95 (− 20.88, 146.78)	0.142
Drug (lithium vs. valproate)	− 7.5 (− 64.9, 49.9)	0.798	− 3.57 (− 81.89, 74.76)	0.929
Baseline eGFR	− 1.03 (− 2.49, 0.43)	0.168	− 2.17 (− 4.15, − 0.20)	0.031
Average daily dose	− 0.03 (− 0.17, 0.1)	0.629	0.02 (− 0.15, 0.18)	0.857
Years on treatment	0.01 (− 5.34, 5.36)	0.997	− 1.47 (− 7.07, 4.14)	0.608
Episodes of lithium toxicity (≥ 1 episode)				
TDM > 0.8 mmol/L	− 5.27 (− 78.89, 68.36)	0.888	2.78 (− 114.5, 120.06)	0.963
TDM > 1.0 mmol/L	− 7.50 (− 182.52, 167.53)	0.933	− 9.05 (− 220.15, 202.06)	0.933
TDM > 1.2 mmol/L	− 7.77 (− 108.68, 93.14)	0.880	1.54 (− 150.36, 153.43)	0.984

**Fig. 2:**
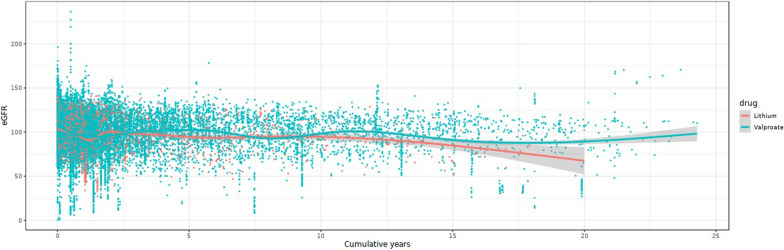
Scatter plot of eGFR over follow-up period

**Fig. 3:**
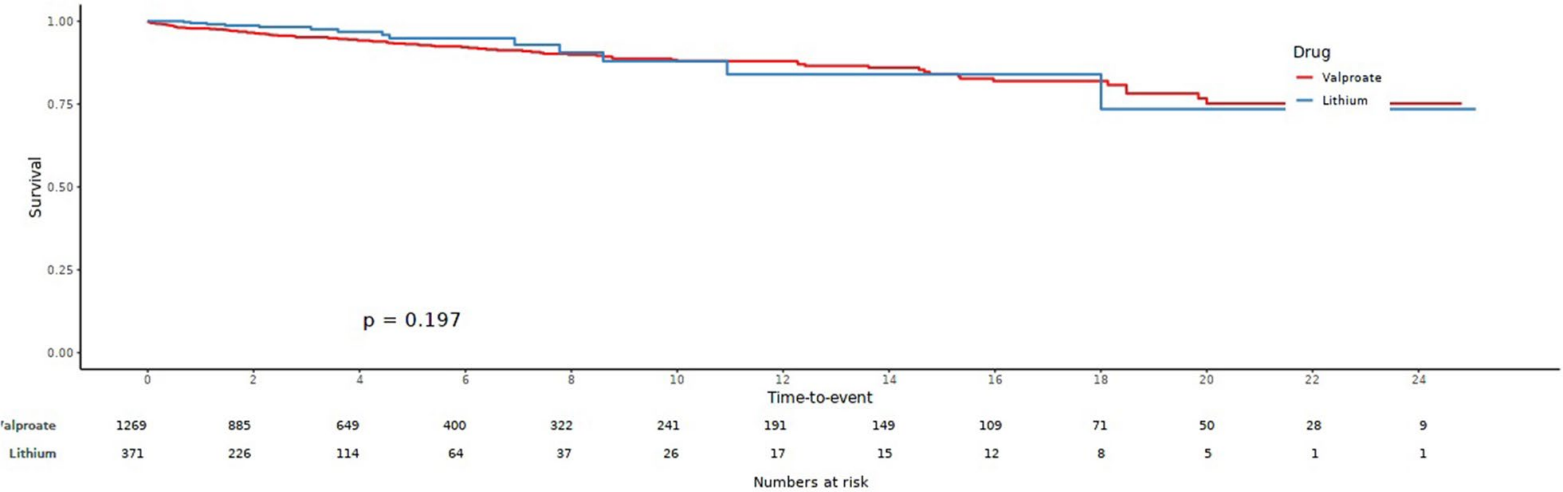
Kaplan–Meier plot on years of treatment taken to enter CKD stage 3 (eGFR  < 60 mL/min/1.73 m^2^)

Figure 2 shows a scatter plot of all eGFR values measured during the follow-up duration for the lithium group (red) and the valproate group (green). While eGFR values of the lithium group generally showed a decreasing pattern with longer duration of treatment, few seemed to fall below significant levels (CKD stages 3–5; eGFR  < 60 mL/min/1.73 m^2^). Figure 3 shows a Kaplan–Meier plot for years of treatment taken to enter CKD stage 3 (eGFR  < 60 mL/min/1.73 m^2^). Although analysis was limited due to a small sample size, the time to enter CKD stage 3 did not show significant differences between lithium and valproate groups (*p * = 0.855).

We additionally compared the basic characteristics between patients finally included in the study and patients excluded due to insufficient eGFR data. Age group and sex were similar between two groups, but the excluded patients from the analyses were more likely to be diagnosed with bipolar disorder (F30–31), were more likely to be on lithium, and had shorter follow-up duration compared to those included in the study. However, among patients excluded in the study, those who had CKD stage 3 were more common in valproate group (lithium group 4.3% vs. valproate group 12.6%, p  = 0.004).

## Discussion

This is the first study that explores the effect of lithium nephrotoxicity in an Asian population. In our study, lithium use did not show greater eGFR decline as compared to valproate use. Kidney function was preserved in both groups up to 15 years of follow-up. As stated earlier, use of diverse psychotropic medication can affect renal function. Polypharmacy is highly prevalent in treatment of bipolar disorder. In particular, valproate and atypical antipsychotics as drugs commonly used as alternatives for lithium are known to have nephrotoxic effects (Gitlin 1999; Hwang et al. 2014). In addition, genetic (Pattaro et al. 2016) and environmental (Barbour et al. 2010a, 2010b) factors both can affect renal function. Thus, having a comparator group that is ethnically identical and likely to be exposed to similar sets of psychotropic medication is crucial in evaluating the nephrotoxicity of lithium. In line with our study findings, Clos et al. (2015) have demonstrated no significant differences in the rate of eGFR decline between those exposed to lithium and those exposed to other first-line drugs after adjusting for various demographic and clinical factors. Our study obtained similar results after adjusting for demographic factors, comorbidities, primary psychiatric diagnoses, and clinical variables such as baseline renal function, treatment duration, and average daily dose of medication.

The relatively lower rate of CKD observed in our study could be associated with ethnic and environmental factors that can affect renal function. Previous studies have suggested that ethnic differences exist in CKD development and that changes in eGFR are associated with genetic variants (Pattaro et al. 2016). Individuals of Asian descent living in western environments have shown increased burden of ESRD and rapid progression of CKD as compared to Caucasians (Barbour et al. 2010a, 2010b), suggesting the effect of gene-environment interaction on renal function. Genetic determinants, alongside exposure to different diet and social environments, may contribute to ethnic differences in CKD progression (Barbour et al. 2010a). Despite such findings, no prior study has reported lithium nephrotoxicity in Asian patients. Further study is needed to confirm our study findings.

Aside from ethnic and environmental differences, specific characteristics of the study population and care settings of our study have to be considered when reviewing our findings. All patients had severe psychiatric illnesses requiring treatment in a tertiary care setting hospital. However, they were well-monitored and were in relatively good physical health. Prior studies with comparator groups have included patients with more frequent medical comorbidities, including hypertension, diabetes, and dyslipidemia, compared to patients in our study (Bocchetta et al. 2013; Clos et al. 2015; Hayes et al. 2016).

It is difficult to compare annual eGFR changes with previous studies directly due to environmental and ethnic differences. The median yearly eGFR change for the lithium maintenance group was − 1.3 mL/min/1.73 m^2^ (IQR: − 6.8, 1.7) in our study. This rate of annual decline was within the normal range of GFR changes (up to 10 mL/min/1.73 m^2^ for 5 years) established by NICE chronic kidney disease guidelines (Crowe et al. 2008). It was not significantly higher than the rate of the comparator group. Previous studies have reported annual rate of eGFR decline to be 1.0–5.0 mL/min/1.73 m^2^ in long-term lithium treatment groups (McKnight et al. 2012; Clos et al. 2015; Bocchetta et al. 2017). Our estimates for annual decline are comparable to previous findings of Clos et al. (2015) (mean annual eGFR decline of 1.3 mL/min/1.73 m^2^, SE: 0.02, t test, *p*  > 0.05).

In our analysis, only 10 (1.3%) participants developed CKD stage 3. The incidence of CKD was not significantly different between the two groups (2 on lithium vs. 8 on valproate, Fisher’s exact test, *p*  = 0.733). No participant reached CKD stages 4–5, which might be due to close monitoring and timely intervention. The prevalence of CKD development was relatively low in our study population, considering that the prevalence of CKD stage 3 in the general population of Korea was 7.9% in the national survey of 2011–12 and the prevalence of CKD stage 3–4 (6.9%) in the US population (Ji and Kim 2016; Murphy et al. 2016), Previous studies in western environments have also reported higher CKD prevalence of 21–55% in long-term lithium users (Lepkifker et al. 2004; Bendz et al. 2010; Tredget et al. 2010; Aiff et al. 2014). The relatively lower age and shorter treatment duration in our study might have affected study findings (vs. mean age of 39.3 ± 16.0 years and mean duration on lithium of 3.5 ± 3.5 years in our study) (Pahwa et al. 2021).

In previous literature, age, female sex, duration of lithium therapy, lower initial eGFR, comorbidities such as hypertension and diabetes, cumulative lithium dose, prior episodes of lithium toxicity, nephrogenic diabetes insipidus and concomitant use of nephrotoxic medication have been identified as factors that can increase the risk of lithium-induced nephropathy (Davis et al. 2018). In our study, baseline eGFR was the only factor that showed a significant correlation with eGFR change in linear regression analyses. Correlation between age and eGFR change was marginally significant (p  = 0.056). Insignificant correlation between episode of lithium toxicity and decreased renal function may have reflected regular monitoring and immediate intervention. These findings need to be further investigated in a larger sample of Korean patients with longer lithium exposure to determine predictors for eGFR decline. Although we could not conduct statistical analysis due to a small sample size, both lithium users who reached CKD stage 3 had comorbid hypertension with toxic serum lithium level  > 1.0 mmol/L at least once.

The strength of this study was that it was based on a well-monitored group of patients with regular follow-up on serum creatinine, eGFR, and serum lithium levels. The hospital-based setting provided abundant clinical data and allowed comprehensive evaluation of each patient. We also selected a comparator group that was likely to be treated under similar circumstances.

However, the present study also has limitations. The tertiary hospital setting might have contributed to selection bias of participants recruited in this study. Excluding those who have been exposed to both drugs also might have led to selection bias. Lithium users are frequently monitored for renal function and serum concentration, leading to the possibility of surveillance bias. As such, patients who receive long-term lithium maintenance therapy are likely to be those with sustained renal function and tolerability to the medication (Nielsen et al. 2018). Frequent monitoring of renal function may have resulted in timely discontinuation in others with less tolerability. In our study, patients who were excluded from the analysis due to insufficient eGFR data had shorter follow-up duration compared to those included in the study. We also did not explore the effect of co-prescribed medications. Discrepancies in the diagnoses have also limited our analysis, as failure to control for diagnosis of bipolar disorder have previously been associated with potential risk of confounding (Nielsen et al. 2018). Diagnostic discrepancies may explain the considerable number of patients diagnosed with schizophrenia or depressive disorder being treated with lithium or valproate in our study. It is notable that lithium use in schizophrenia and depression are quite common (Lim et al. 2020; Vazquez et al. 2021), and that diagnostic changes between schizophrenia, bipolar disorder and depression are also common (Kim et al. 2011). This study was based on a limited number of patients, and thus needs to be replicated with a larger sample of Asian population.

## Conclusions

There was little evidence that Korean patients treated with lithium developed significant decline in renal function more rapidly than patients treated with valproate. In light of these findings, lithium is likely to be safe for long-term use with careful monitoring and intervention in majority of patients.

## Supplementary Information

**Additional file 1: Table S1.** Characteristics of two lithium patients who reached CKD stage 3. **Table S2-1.** Linear regression predicting yearly eGFR change, conducted as subgroup analysis in patients diagnosed with F20–29. **Table S2-2.** Linear regression predicting yearly eGFR change, conducted as subgroup analysis in patients diagnosed with F30–31. **Table S2-3.** Linear regression predicting yearly eGFR change, conducted as subgroup analysis in patients diagnosed with F32–33.

## Data Availability

The dataset are available from the corresponding author upon reasonable request.

## Abbreviations

eGFR: Estimated glomerular filtration rate
CKD: Chronic kidney disease
ESRD: End-stage renal disease
CKD-EPI: Chronic kidney disease epidemiology collaboration
