# Supplementary material for: Estimated glomerular filtration rate in Korean patients exposed to long-term lithium maintenance therapy
Source: Int J Bipolar Disord. 2022 Feb 7;10:4. doi: 10.1186/s40345-022-00249-5 (PMC8818587; doi:10.1186/s40345-022-00249-5)
Supplement: Supplementary file 1 — Additional file 1: Table S1. Characteristics of two lithium patients who reached CKD stage 3. Table S2-1. Linear regression predicting yearly eGFR change, conducted as subgroup analysis in patients diagnosed with F20–29. Table S2-2. Linear regression predicting yearly eGFR change, conducted as subgroup analysis in patients diagnosed with F30–31. Table S2-3. Linear regression predicting yearly eGFR change, conducted as subgroup analysis in patients diagnosed with F32–33. [file 40345_2022_249_MOESM1_ESM.docx]

**Table S1** Characteristics of two lithium patients who reached CKD stage 3

|  | **Patient 1** | **Patient 2** |
| --- | --- | --- |
| Age | 53 | 64 |
| Sex | Male | Female |
| Comorbidities | Hypertension  Parkinsonism  Ischemic stroke | Hypertension  Cerebral infarction |
| Primary psychiatric diagnoses, ICD-10 | F31 (Bipolar disorder) | F33 (Depressive disorder) |
| Average TDM | 0.882 mmol/L | 1.06 mmol/L |
| Average daily lithium dose | 641.24 mg | 298.86 mg |
| Episode of lithium toxicity | Lithium TDM >1.0 mmol/L | Lithium TDM >1.0 mmol/L |
| Duration from lithium initiation to CKD development | 15.88 years | 9.82 years |
| Cumulative duration of lithium treatment | 15.78 years | 2.41 years |
| Baseline eGFR | 79.13 mL/min/1.73m^2^ | 62 mL/min/1.73m^2^ |
| Year 1 eGFR | 72.68 mL/min/1.73m^2^ | 60.8 mL/min/1.73m^2^ |
| Second to last eGFR | 51.79 mL/min/1.73m^2^ | 49 mL/min/1.73m^2^ |
| Last eGFR | 51.56 mL/min/1.73m^2^ | 39 mL/min/1.73m^2^ |

**Table S2-1** Linear regression predicting yearly eGFR change, conducted as subgroup analysis in patients diagnosed with F20-29

|  | **Crude coefficient**  **(95%CI)** | **Crude *p*** | **Adjusted coefficient**  **(95%CI)** | **Adjusted *p*** |
| --- | --- | --- | --- | --- |
| Age | 0.02 (-0.2,0.24) | 0.856 | -0.04 (-0.34,0.26) | 0.795 |
| Sex (men vs women) | -2.86 (-8.72,3) | 0.341 | -3.53 (-9.97,2.91) | 0.285 |
| Baseline hypertension | -9.12 (-21.2,2.95) | 0.141 | -7.71 (-21.28,5.87) | 0.268 |
| Baseline diabetes | 0.5 (-15.54,16.53) | 0.951 | 4.77 (-11.88,21.42) | 0.575 |
| Drug (lithium vs valproate) | -1.39 (-7.98,5.2) | 0.68 | -5.85 (-14.93,3.23) | 0.209 |
| Baseline eGFR | -0.11 (-0.28,0.06) | 0.219 | -0.14 (-0.36,0.08) | 0.215 |
| Average daily dose | 0 (-0.01,0.02) | 0.702 | 0.01 (-0.02,0.03) | 0.592 |
| Years on treatment | 0.14 (-0.43,0.7) | 0.641 | 0.02 (-0.59,0.63) | 0.953 |
| Episodes of lithium toxicity (≥1 episode) | | | | |
| TDM > 0.8 mmol/L | 0.58 (-13.07,14.23) | 0.934 | -5.73 (-24.97,13.51) | 0.56 |
| TDM > 1.0 mmol/L | 1.28 (-23.81,26.36) | 0.921 | 0.15 (-30.04,30.33) | 0.992 |
| TDM > 1.2 mmol/L | 0.02 (-0.2,0.24) | 0.856 | -0.04 (-0.34,0.26) | 0.795 |

**Table S2-2** Linear regression predicting yearly eGFR change, conducted as subgroup analysis in patients diagnosed with F30-31

|  | **Crude coefficient**  **(95%CI)** | **Crude *p*** | **Adjusted coefficient**  **(95%CI)** | **Adjusted *p*** |
| --- | --- | --- | --- | --- |
| Age | 0.82 (-0.88,2.53) | 0.343 | 0.37 (-2.1,2.84) | 0.770 |
| Sex (men vs women) | 17.23 (-34.77,69.24) | 0.516 | 17.28 (-38.7,73.25) | 0.546 |
| Baseline hypertension | 5.1 (-93.82,104.02) | 0.92 | -9.27 (-117.41,98.87) | 0.867 |
| Baseline diabetes | 19.31 (-127.21,165.82) | 0.796 | 5.54 (-155.25,166.32) | 0.946 |
| Drug (lithium vs valproate) | 24.82 (-28.2,77.85) | 0.359 | 30.89 (-40.25,102.03) | 0.395 |
| Baseline eGFR | -0.99 (-2.41,0.42) | 0.17 | -0.7 (-2.67,1.27) | 0.486 |
| Average daily dose | 0.02 (-0.11,0.15) | 0.772 | -0.01 (-0.16,0.14) | 0.890 |
| Years on treatment | 3.38 (-2.69,9.45) | 0.276 | 3.05 (-3.37,9.47) | 0.352 |
| Episodes of lithium toxicity (≥1 episode) | | | | |
| TDM > 0.8 mmol/L | 18.93 (-45.13,83) | 0.563 | 3.25 (-102.14,108.65) | 0.952 |
| TDM > 1.0 mmol/L | 13.7 (-69.34,96.74) | 0.747 | -4.42 (-132.5,123.66) | 0.946 |
| TDM > 1.2 mmol/L | 12.63 (-128.74,154.01) | 0.861 | -11.61 (-184.95,161.74) | 0.896 |

**Table S2-3** Linear regression predicting yearly eGFR change, conducted as subgroup analysis in patients diagnosed with F32-33

|  | **Crude coefficient**  **(95%CI)** | **Crude *p*** | **Adjusted coefficient**  **(95%CI)** | **Adjusted *p*** |
| --- | --- | --- | --- | --- |
| Age | -4.00 (-9.5,1.5) | 0.156 | -9.71 (-17.94,-1.48) | 0.022 |
| Sex (men vs women) | -79.51 (-268.62,109.61) | 0.411 | -100.95 (-296.64,94.75) | 0.314 |
| Baseline hypertension | -61.34 (-340.85,218.18) | 0.668 | 13.33 (-327.49,354.15) | 0.939 |
| Baseline diabetes | -48.36 (-519.43,422.72) | 0.841 | 147.78 (-375.98,671.54) | 0.581 |
| Drug (lithium vs valproate) | -65.06 (-324.75,194.64) | 0.624 | -104.06 (-454.72,246.61) | 0.562 |
| Baseline eGFR | -0.95 (-6.18,4.28) | 0.722 | -6.72 (-13.77,0.33) | 0.064 |
| Average daily dose | 0.04 (-0.59,0.68) | 0.892 | 0.05 (-0.64,0.74) | 0.895 |
| Years on treatment | -4.92 (-20.17,10.33) | 0.528 | -11.73 (-28.43,4.97) | 0.171 |
| Episodes of lithium toxicity (≥1 episode) | | | | |
| TDM > 0.8 mmol/L | -57.41 (-768.46,653.64) | 0.874 | -85.55 (-1175.31,1004.22) | 0.878 |
| TDM > 1.0 mmol/L | -59.56 (-1283.97,1164.84) | 0.924 | 145.48 (-1380.35,1671.3) | 0.852 |
| TDM > 1.2 mmol/L | -4 (-9.5,1.5) | 0.156 | -9.71 (-17.94,-1.48) | 0.022 |
